# Supplementary material for: An ontogeny-cytokine code determines macrophage response polarity and tumor outcomes
Source: Commun Biol. 2026 Mar 13;9:592. doi: 10.1038/s42003-026-09853-y (PMC13129106; doi:10.1038/s42003-026-09853-y)
Supplement: Supplementary file 2 — Supplementary Information [file 42003_2026_9853_MOESM2_ESM.pdf]

An ontogeny-cytokine code determines macrophage response polarity and tumor outcomes

Dominik J. Schaer[1], Nadja Schulthess-Lutz[1], Matthias J. Peterhans[1], Livio Baselgia[1], Melanie Eschment[1], Rok Humar,[1] Florence Vallelian*[1]

[1] Department of Internal Medicine, University Hospital and University of Zurich, Zurich, Switzerland

Supplementary Figures: Pages 2-7

Materials and Resources: Pages 8-9

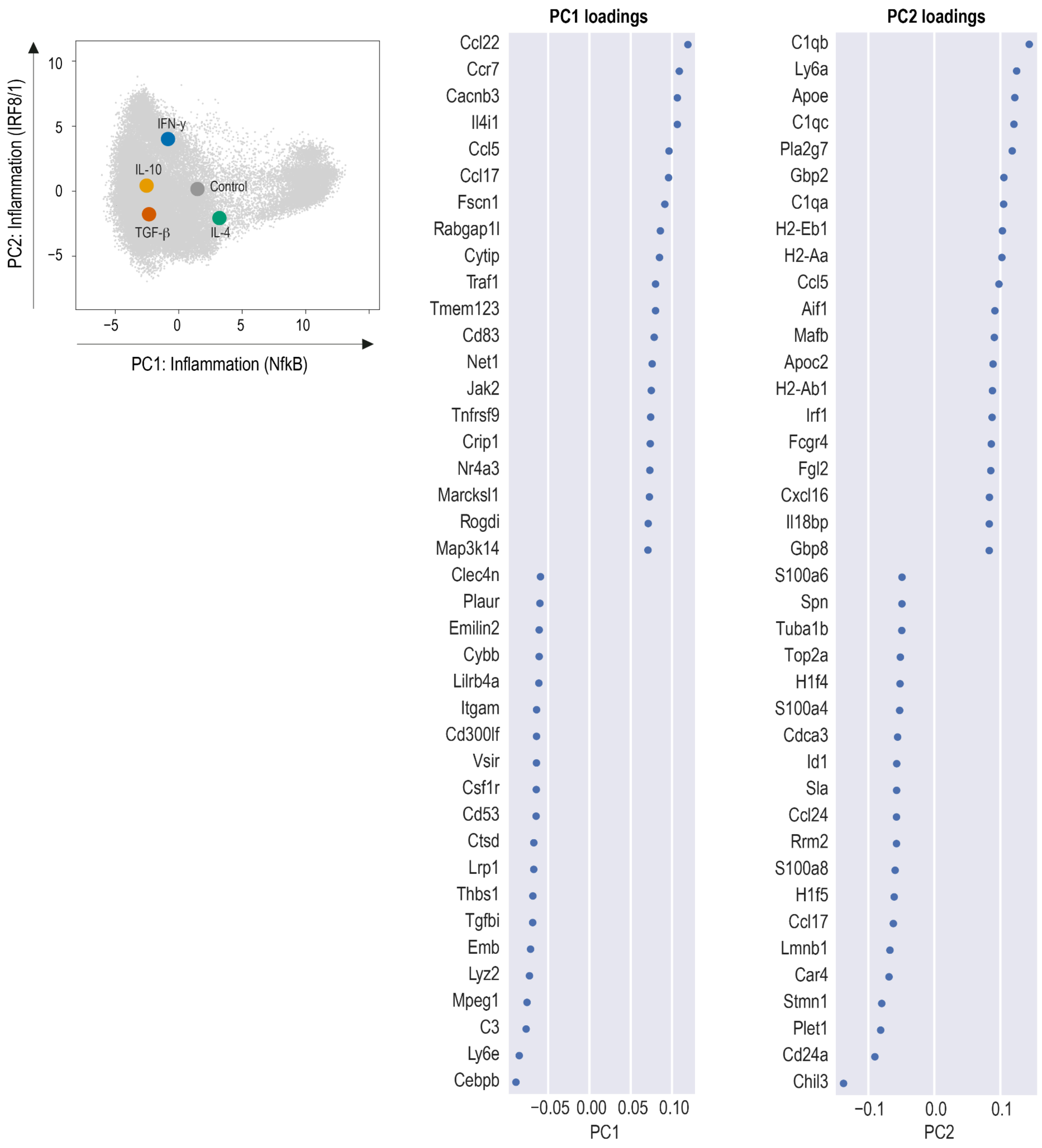

Supplementary Figure 1

BM precursors were cultured with GM-CSF alone or in combination with IFN-γ, IL-4, IL-10, or TGF-β and analyzed by multiplexed scRNA-seq (see Fig. 2). Principal-component analysis (PCA) identified two dominant axes of variation. The 20 genes with the highest positive and negative loadings for PC1 and PC2 are listed. Each dot represents one gene; x-axis shows PC loading.

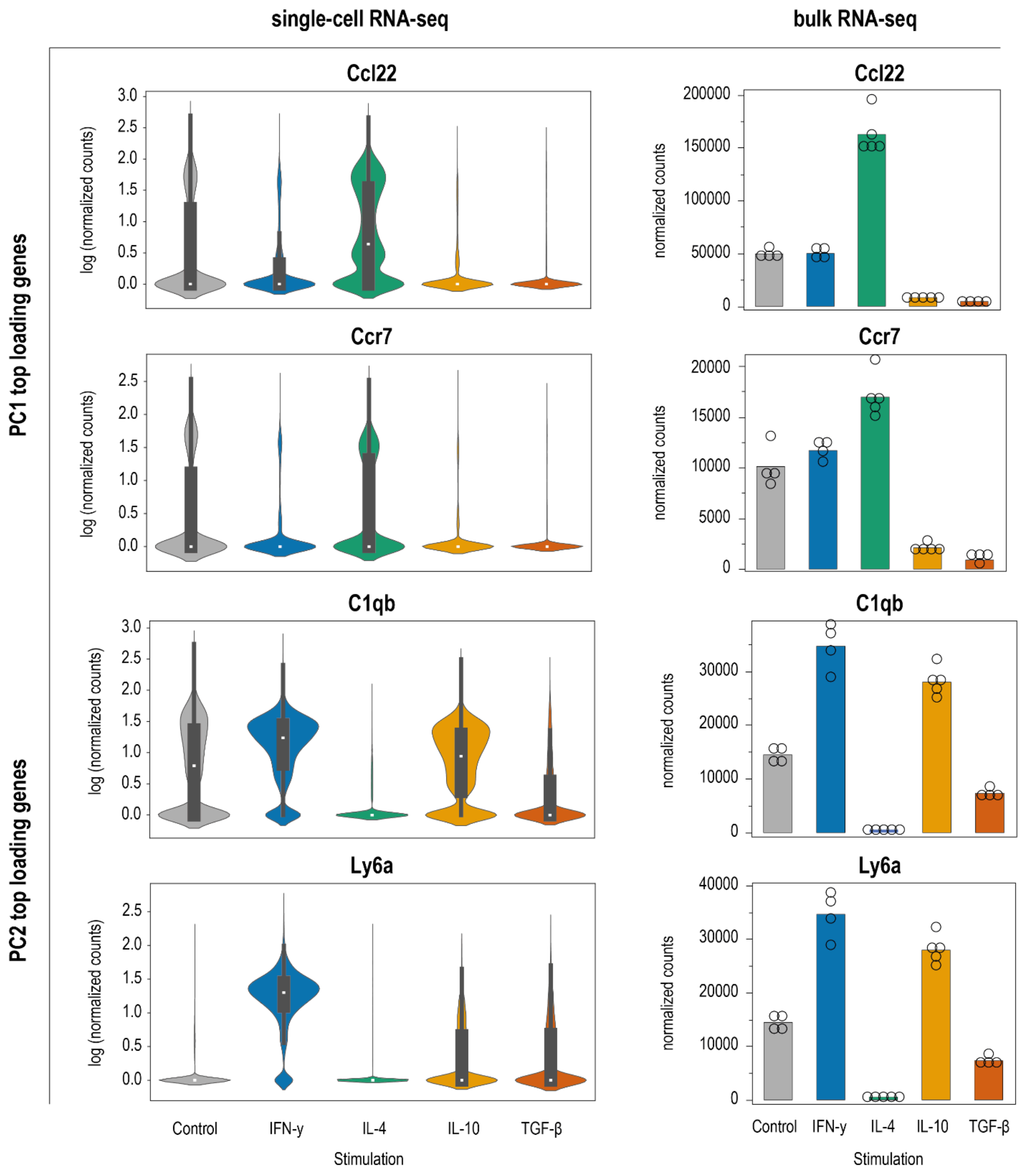

Supplementary Figure 2

Left panels: Violin plots showing distribution of the log-normalized count expression for the two top positive loading genes from PC1 and PC2 in the scRNA seq data set of GM-CSF macrophages (see Figure 2). Each violin plot represents one cytokine condition. Violin width indicates the density of cells at each expression level; white dots show median values. Right panels: Independent validation by bulk RNA-seq showing normalized counts for the same genes. Bar represents the mean of n= 5 biological replicates.

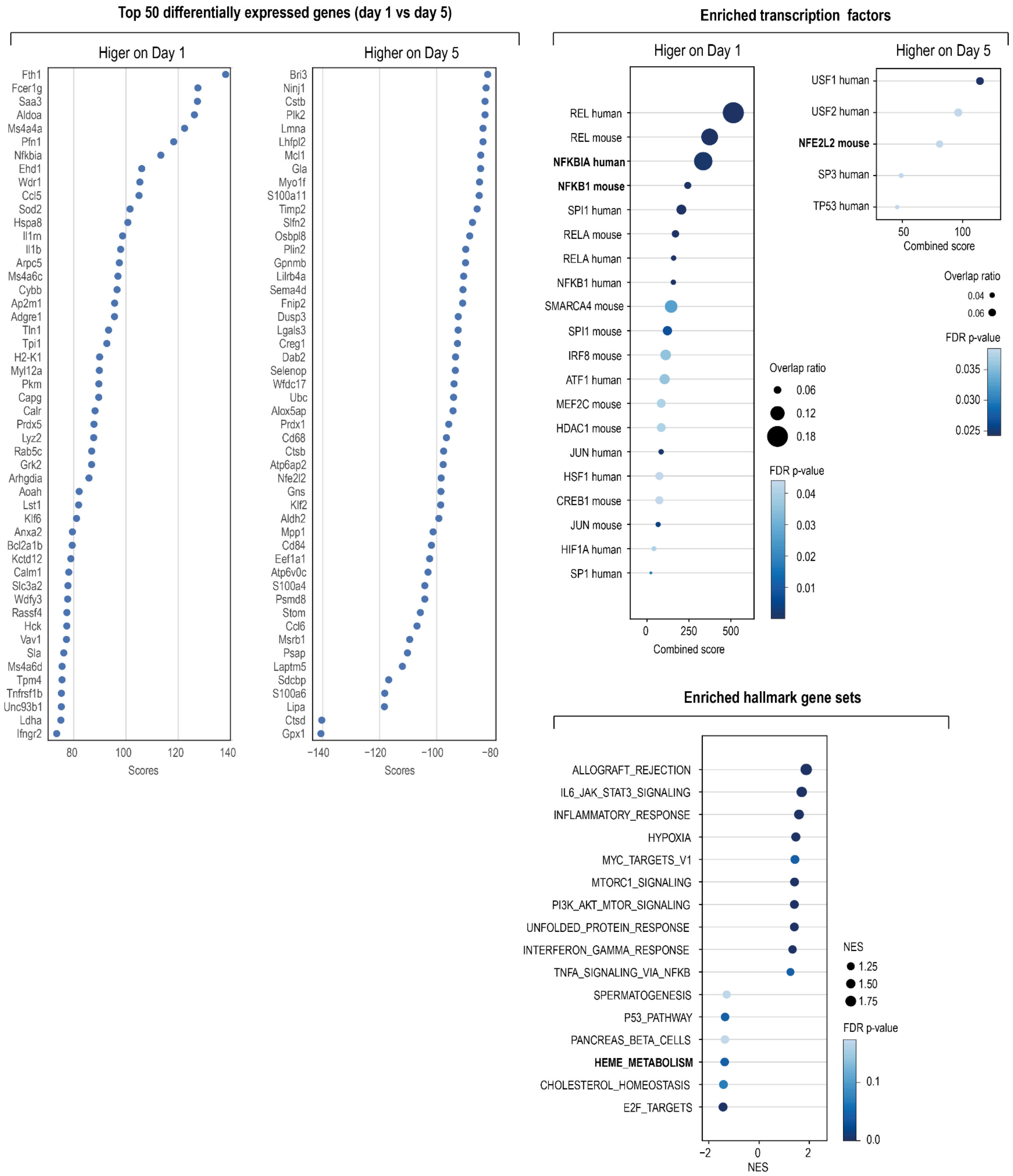

Supplementary Figure 3

Time-resolved, multiplexed scRNA-seq experiment of mixed cell spheroids composed of M-CSF macrophages and MC38 cancer cells (see Fig. 4).

Left panels: Top 50 differentially expressed genes. Dot plots showing the top 25 upregulated genes at day 1 (positive, top) and top 25 upregulated genes at day 5 (negative, bottom) in macrophages. Each dot represents a single gene; the x-axis shows mean normalized expression (log scale).

Right panels: GSEA using transcription factor enrichment and MSigDB Hallmark gene sets. Dot size represents overlap ratio; color intensity (blue scale) represents combined enrichment score (integrating overlap and statistical significance, with FDR p-value threshold shown).

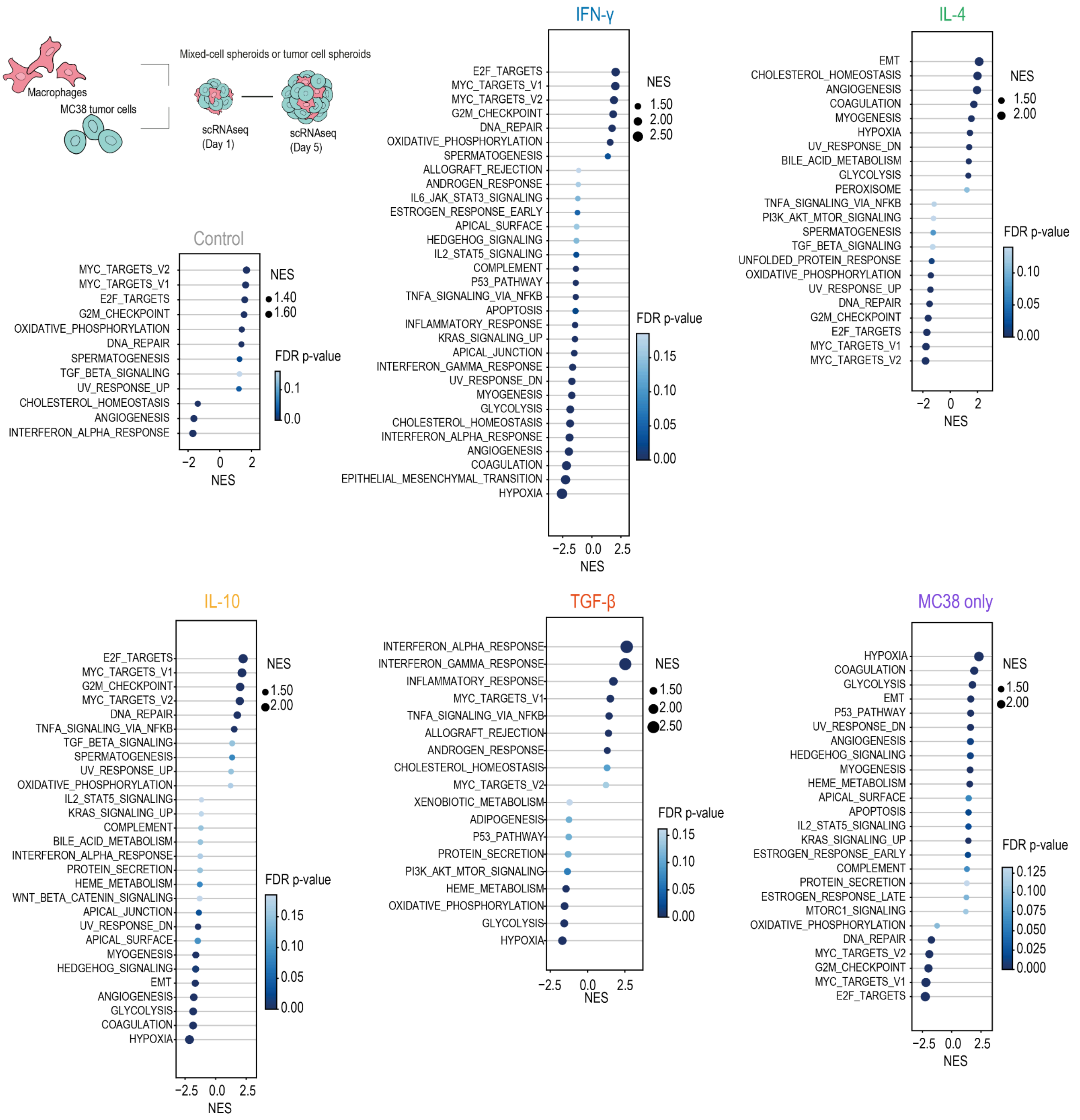

Supplementary Figure 4

Time-resolved, multiplexed scRNA-seq experiment of mixed cell spheroids composed of M-CSF macrophages and MC38 cancer cells (see Fig. 4). Cells per treatment were functionally annotated according to GSEA using MSigDB Hallmark gene sets.

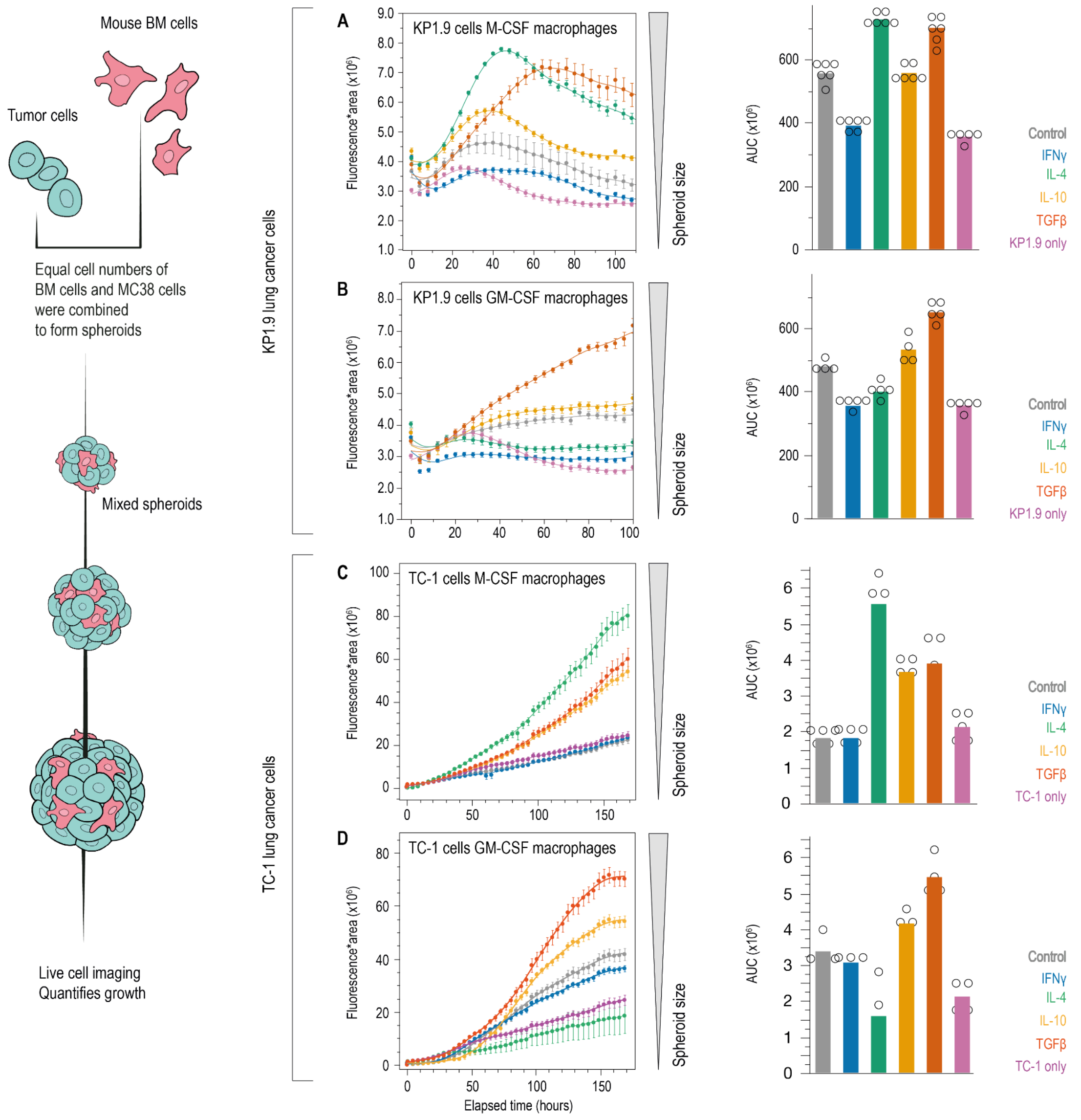

Supplementary Figure 5

A. GFP-KP1.9 cells were cultured alone or mixed 1:2 with cytokine-polarised M-CSF or GM-CSF macrophages in ultra-low-attachment plates and imaged for five days.

M-CSF macrophages: Integrated GFP fluorescence intensities across the spheroid area over time, demonstrating enhanced spheroid growth in the presence of anti-inflammatory macrophages. Data are the mean ± SE of 4-6 replicates analyzed within one representative experiment. AUC quantification, one-way ANOVA Dunnett posttest corrected for multiple comparisons (control vs IFN-γ p <0.0001, control vs IL-4 p<0.0001, control vs IL-10 p >0.999 (ns), control vs TGF-β p<0.0001, control vs TC <0.0001).

B. GM-CSF macrophages: Time-course analysis and AUC quantification identical to panel A (Control vs IFN-γ p<0.0001, control vs IL-4 p 0.0007, control vs IL-10 p 0.0376, control vs TGF-β p<0.0001, control vs TC p<0.0001).

C. mScarlet TC-1 cells were cultured alone or mixed 1:1 with cytokine-polarised M-CSF or GM-CSF macrophages in ultra-low-attachment plates and imaged for seven days.

M-CSF macrophages: Integrated red fluorescence intensities across the spheroid area over time, demonstrating enhanced spheroid growth in the presence of anti-inflammatory macrophages. Data are the mean ± SE of 4-5 replicates analyzed within one representative experiment. AUC quantification, one-way ANOVA Dunnett posttest corrected for multiple comparisons (control vs IFN-γ $p > 0.99$ (ns) , control vs IL-4 $p<0.0001$, control vs IL-10 $p<0.0001$, control vs TGF-β $p<0.0001$, control vs TC $p = 0.74$).

D. GM-CSF macrophages: Time-course analysis and AUC quantification identical to panel AC (Control vs IFN-γ $p = 0.87$, control vs IL-4 $p = 0.0006$, control vs IL-10 $p = 0.1813$, control vs TGF-β p 0.0001, control vs TC $p = 0.0146$).

Materials and Resources

Mouse strain	Company
C57BL/6	Charles River
B6.Cg-Tg(TcraTcrb)425Cbn/J (OT-2)	Swiss Immunological Mouse repository (SwImMR)
VavCre	Swiss Immunological Mouse repository (SwImMR)
Ai14tdTomato	Jackson Laboratory Strain #:007914
Arg1-ires-YFP	Jackson Laboratory Strain #:015857
Spp1-IRES-tdTomato	Jackson Laboratory Strain #:033731

Tumor lines	Company
GFP+ MC38 colon adenocarcinoma cells	Donated by Gerhard Christofori, Department of Biomedicine, University of Basel
KP1.9	Group Mikael Pittet University of Geneva

Antibodies for flow cytometry

Targeted antigen	Clone	Fluorochrome	Species	Company	Product number
CD4	GK1.5	Phycoerythrin (F	Rat	Biolegend	100408
I-A/I-E	M5/114.15.2	Brilliant violet 42	Rat	BD	562564
CD11b	M1/70	APC-Cy7	Rat	Biolegend	101226
Rat IgG2b, λ Isotype Ctrl Antibody	G013B8	Phycoerythrin (F	Rat	Biolegend	403804
BV421 Rat IgG2b, κ Isotype Control	R35-38	Brilliant violet 42	Rat	BD	562603
APC/Cyanine7 Rat IgG2b, κ Isotype Ctrl Ant	RTK4530	APC-Cy7	Rat	Biolegend	400624

Reagents for in vivo or in vitro treatments

Product name	Company	Product number
Ketamine	Graeub	QN01AX03
Xylazine	Bayer	QN05CM92
Acepromazine	Fatro	QN05AA04
Mouse IL-4 Recombinant Protein, PeproTec	Thermo Fisher	214-14-100UG
Mouse IFN-gamma Recombinant Protein, Pe	ThermoFisher	315-05-100UG
Human TGF-beta 1 Recombinant Protein, P	ThermoFisher	100-21-10UG
Human IL-10 Recombinant Protein, PeproTe	ThermoFisher	200-10-100UG

Magnetic beads, magnets and associated antibodies

Product name	Company	Product number
Dynabeads™ FlowComp™ Mouse CD4 Kit	Invitrogen	11461D

Solutions, medium, supplement and enzymes

Product name	Company	Product number
Phosphate buffered Saline (PBS)	Gibco	10010-015
L-glutamine 200mM (100x)	Gibco	25030-024
RPMI Medium	Gibco	11835-063
Dulbecco's MEM	Merck	1469C
Fetales bovines Serum	Sigma-aldrich	S0615
MEM NEAA (100x)	Gibco	11140-035
Sodium-Pyruvate	Sigma	P2256
Penicillin-Streptomycin	Thermo Fisher	15140-122
Liberase	Roche	5401119001
Dnase I	Roche	10269638001
UltraPure™ 0.5M EDTA, pH 8.0	ThermoFisher	15575020
MACS Buffer BSA Stock Solution	Miltenyi Biotec	130-091-376
20% Glucosum	Bichsel	FE1001327

Peptides, and Recombinant proteins

Product name	Company	Product number
Ovalbumin (323-339) (chicken, Japanese qu	Sigma Aldrich	O1641-5MG
Recombinant Murine M-CSF	Peprotech	315-02
Recombinant Murine GM-CSF	Peprotech	315-03

Chemicals

Product name	Company	Product number
Formalin 10%, gepuffert	Formafix	01-1061
Hematoxylin solution acc. to Gill II	Carl Roth	T864.2
Eosin Y w/ Phloxine solution	Epredia	71304

Critical Commercial Assays

Product name	Company	Product number
UltraComp eBeads™ Compensation Beads	Thermo Fisher	01-2222-42
Tru Stain FcxTmPLUS CD16/32, clone S17(	Biolegend	156604
GEM-X Flex Sample Preparation v2 Kit, 48 r	10x Genomics	1000781
GEM-X Flex Gene Expression Chip Kit, 4 ch	10x Genomics	1000791
GEM-X Flex Gene Expression Mouse 4-plex	10x Genomics	1000797
Chromium Next GEM Single Cell Fixed RNA	10x Genomics	1000414
Chromium Next GEM Chip Q Single Cell Kit,	10x Genomics	1000422
Dual Index Kit TS Set A, 96 rxn	10x Genomics	1000251
Chromium Fixed RNA Kit, Human Transcripto	10x Genomics	1000475
RNA screentape	Agilent Technologies	5067-5576
RNA ScreenTape Sample Buffer	Agilent Technologies	5067-5577
RNA ScreenTape Ladder	Agilent Technologies	5067-5578
Genomic DNA screentape	Agilent Technologies	5067-5365
Genomic DNA Reagents	Agilent Technologies	5067-5366
SYBR™ Green Master Mix	Applied Biosystems	4385612
Illumina® Stranded mRNA Prep, Ligation (96	Illumina	20040534
RNeasy Mini Kit	Qiagen	74106
CellTrace™ Far Red Cell Proliferation Kit, for	Thermo Fisher	C34564

Plates and coating

Product name	Company	Product number
96-well plates	Techno Plastic Products (TPP)	92096
12-well plates	Techno Plastic Products (TPP)	92012
6-well plates	Techno Plastic Products (TPP)	92006
6 cm culture dish	Techno Plastic Products (TPP)	93100
15 cm culture dish	Techno Plastic Products (TPP)	93150
Nunc™ Multidishes with UpCell™ Surface 6v	Thermo Fisher	174901
Nunc™ Dishes with UpCell™ Surface; 60mm	Thermo Fisher	174903
PrimeSurface® 3D culture Ultra-low Attachm	S-Bio	MS-9096UZ
SphericalPlate® 5D microwell, 24 well	Axon-Lab	12038828
96-well Clear Round Bottom Not Treated Mic	Falcon®	351177
