# Supplementary material for: An ontogeny-cytokine code determines macrophage response polarity and tumor outcomes
Source: Commun Biol. 2026 Mar 13;9:592. doi: 10.1038/s42003-026-09853-y (PMC13129106; doi:10.1038/s42003-026-09853-y)
Supplement: Supplementary file 3 — Description of Additional Supplementary files [file 42003_2026_9853_MOESM3_ESM.pdf]

## Description of Additional Supplementary files

File name: Supplementary Data 1

Description: Source data underlying all graphs and charts in the main figures are provided as Supplementary Data 1
